# Better Late Than Never

**DOI:** 10.1016/j.jaccas.2023.101750

**Published:** 2023-03-15

**Authors:** Arooj Khan, Eric Heller, Grace Alexander, James Hopson

**Affiliations:** University of Iowa Hospitals and Clinics, Iowa City, Iowa, USA

**Keywords:** bradycardia, sinus node dysfunction, ventricular escape

## Abstract

Symptomatic bradycardia may be iatrogenic or from a conduction system abnormality. Here we show an interesting case of iatrogenic symptomatic bradycardia that may be confused with a conduction system abnormality. (**Level of Difficulty: Advanced.**)

## Central Illustration

**Figure:**
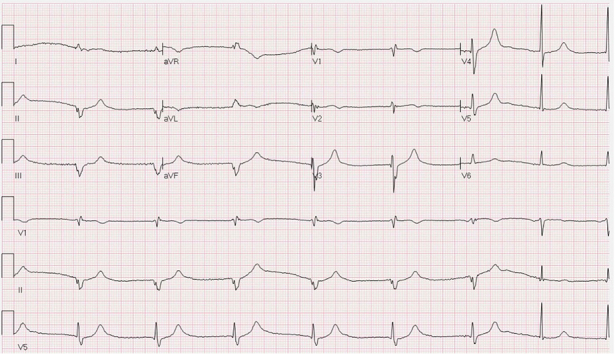

**Figure 1:**
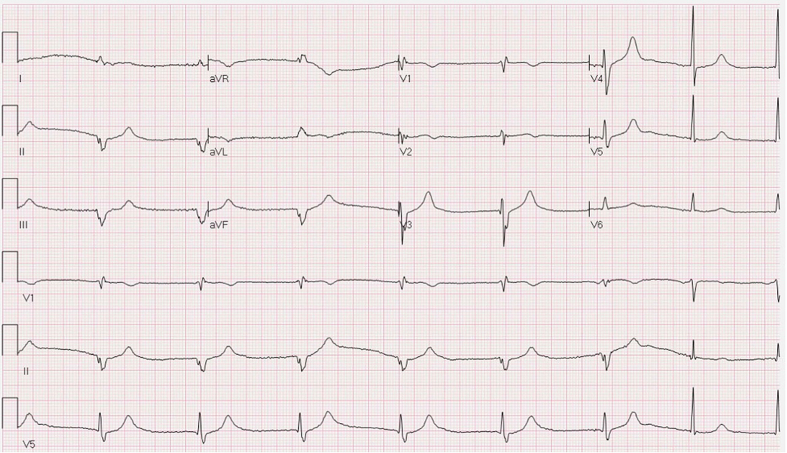
Electrocardiography on Presentation to Clinic

An 81-year-old White man has a history of heart failure with preserved ejection fraction, hypertension, hyperlipidemia, and chronic kidney disease. Relevant medications include metoprolol succinate. Amlodipine was changed to diltiazem after an emergency department visit 6 weeks earlier with atrial fibrillation and rapid ventricular response. He now presents to the clinic for 3 weeks’ history of fatigue and activity intolerance. Vital signs show heart rate of 43 beats/min, blood pressure of 158/78 mm Hg, and oxygen saturation of 94% on room air. His cardiac examination was unremarkable except for bradycardia; laboratory test results were normal except for creatinine (1.6 mg/dl). An electrocardiography (ECG) was obtained and is shown in Figure 1.

This ECG shows:

A. Atrial fibrillation with slow rate and a late return of conducted sinus bradycardia
B. Bradycardia with a junctional escape and late return of conducted sinus bradycardia
C. Bradycardia with a ventricular escape and late return of conducted sinus bradycardia
D. Intermittent atrioventricular block with ventricular escape

## Discussion/Rationale

The correct answer is C. The source of the symptoms is bradycardia, and the source of the bradycardia is prominent sinus bradycardia, perhaps medication related. The start of the ECG finds a ventricular escape rhythm without visible sinus activity. A hint of sinus activity might precede the fifth beat but is clearly present on the sixth, and this sixth QRS can be seen to be a fusion beat. This identifies the escape as being ventricular, not junctional. (As a side note: the QRS morphology argues for the escape to be in the left posterior fascicle.) There is no atrioventricular block or atrial fibrillation on the tracing. We would argue that the same medication issues that led to sinus node dysfunction may have also impaired what might otherwise have been a junctional escape. In this patient’s case, a wandering atrial pacemaker with heart rate in the low 70s (beats/min) (Supplemental Figure 1), indirectly pointing toward sinus node fragility, was seen with the cessation of diltiazem. A pacemaker will be considered if more aggressive atrial fibrillation medication is needed.

## Funding Support and Author Disclosures

The authors have reported that they have no relationships relevant to the contents of this paper to disclose.

