# Supplementary figures and images for: Better Late Than Never
Source: JACC Case Rep. 2023 Mar 15;10:101750. doi: 10.1016/j.jaccas.2023.101750 (PMC10039381; doi:10.1016/j.jaccas.2023.101750)

**Supplemental Figure 1: ECG after diltiazem was discontinued.**


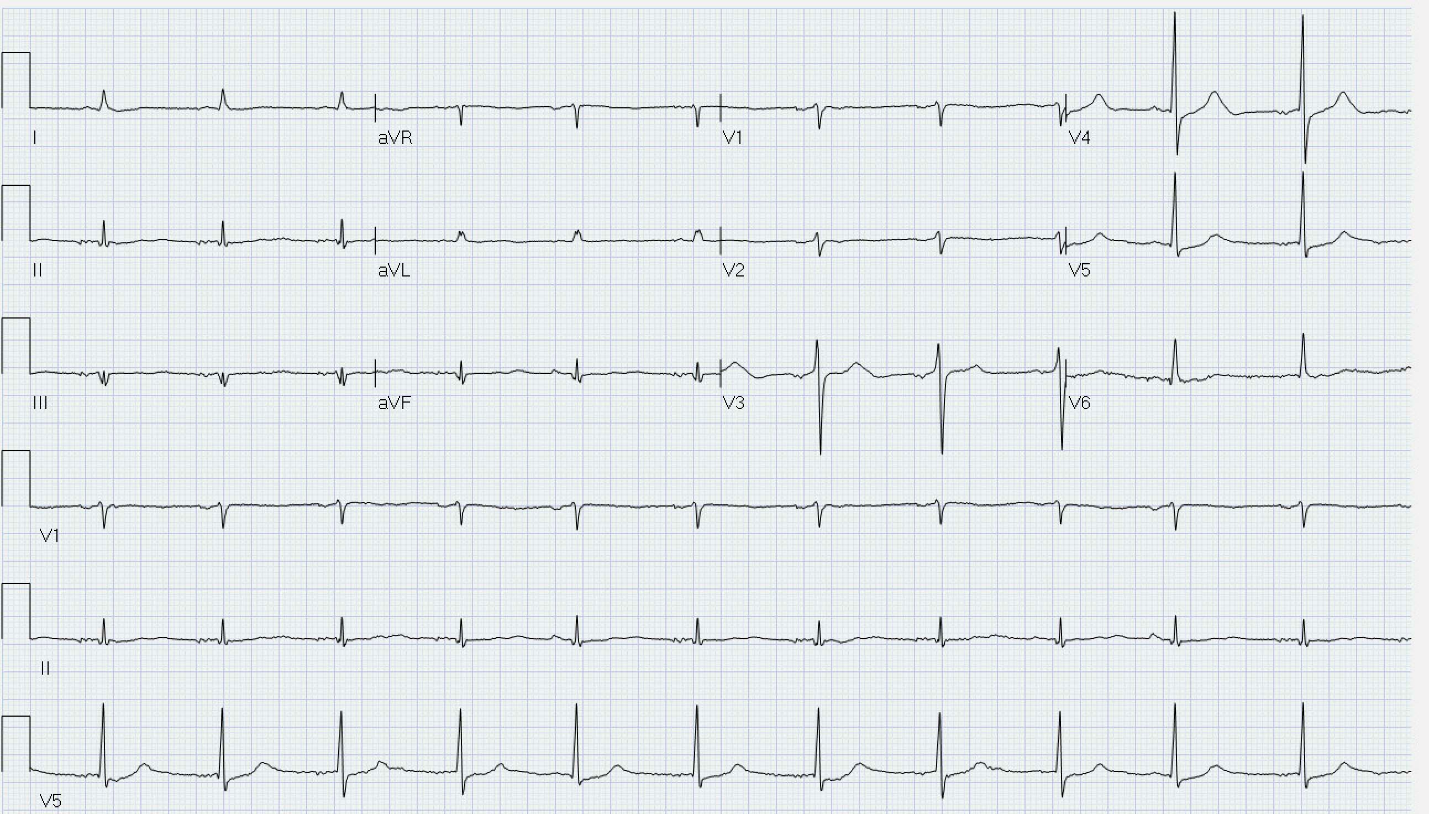

Supplement: Supplemental Figure 1 [file mmc1.docx]
